# Urinary tract endometriosis: Revisiting the definition of ureterolysis

**DOI:** 10.1002/ijgo.70290

**Published:** 2025-07-09

**Authors:** Constance Durant Des Aulnois, Léo Razakamanantsoa, Adrien Crestani, Ylan Abrahami, Steeve Doizi, Florie Gomez, Cyril Touboul, Isabelle Thomassin Naggara, Emile Daraï, Yohann Dabi

**Affiliations:** ^1^ Department of Gynecology‐Obstetrics and Reproductive Medicine, Hôpital Tenon Sorbonne University Paris France; ^2^ Department of Radiology, Hôpital Tenon Sorbonne University Paris France; ^3^ Department of Urology, Hôpital Tenon Sorbonne University Paris France; ^4^ Clinical Research Group (GRC6), Centre Expert en Endométriose (C3E) Sorbonne University Paris France

**Keywords:** bladder endometriosis, endometriosis, surgical management, ureteral endometriosis, ureterolysis, urinary tract endometriosis

## Abstract

**Objectives:**

To report the incidence of urinary tract endometriosis (UTE) among patients with deep endometriosis, and to analyze surgical procedures, the rate of complications, and the recurrence rate in patients with UTE managed in a French expert center.

**Methods:**

We conducted a retrospective analysis of patients treated in the gynecologic surgery department of Tenon Hospital (AP‐HP, Paris, France) between January 1, 2016, and December 31, 2022. Patients that underwent partial bladder resection, extensive and complex ureterolysis, or ureteral resection for deep infiltrating endometriosis were selected. We describe surgical approach, type of intervention on urinary tract lesions, associated gynecologic and digestive procedures, and peri‐ and postoperative complications.

**Results:**

Among 923 patients treated for deep endometriosis, 99 (10.7%) had urinary tract lesions. Bladder surgery was performed in 43 cases (43.4%), including 41 partial cystectomies (41.4%). Ureter procedures were performed in 86 cases (86.9%), including 70 (70.7%) extensive ureterolysis and 16 (16.2%) partial ureteral resections followed by ureteroneocystostomy. Ninety‐three patients (94%) underwent standard or robot‐assisted laparoscopy. Complications requiring surgical reintervention occurred in 17 cases (17.2%). At 1 month postsurgery, 85% of the patients reported not needing analgesics. During follow up, three patients experienced deep endometriosis recurrence (3%).

**Conclusion:**

Patients with UTE can safely undergo conservative management, with an acceptable complication rate. These patients should be managed in referral centers to favor multidisciplinary approaches, including appropriate preoperative workup.

## 1 INTRODUCTION

Endometriosis is characterized by the presence of endometrial‐like tissue outside the uterus.^1^ The prevalence of endometriosis in the general female population is estimated to 3%–10%^2^ and can reach 35%–50% in infertile women.^3^, ^4^ Three endometriosis phenotypes are classically distinguished; the peritoneal superficial phenotype, the endometrioma, and the deep endometriosis affecting about 20% of women with endometriosis and representing the most severe form of the disease.^5^ As defined by Koninckx et al., deep endometriosis refers to endometrial tissue penetrating deeply into organs, and urinary tract endometriosis (UTE) could be considered either as a complication or extension of the lesions.^6^

Among deep endometriosis lesions, UTE alone remains rare, observed in 1%–5.5% of cases mainly involving the bladder (85%), and the ureter (10%), while kidney (4%), and urethra (2%) involvement is less frequent.^7^, ^8^, ^9^, ^10^ Bladder endometriosis is more often localized on the bladder dome (66% of cases).^11^, ^12^ Whereas, the impact of deep endometriosis on ureteral anatomy and function is observed in up to 100% of patients with colorectal endometriosis, representing the most severe form of deep endometriosis.^13^ Serious complications can occur such as hydronephrosis and loss of renal function, often asymptomatic or during surgery, with ureteral injury requiring at least a JJ stent.^8^, ^9^, ^14^, ^15^, ^16^ In 2017, Cavaco‐Gomes et al. produced a systematic review of 18 cohort studies analyzing the laparoscopic management of ureteral endometriosis.^16^ Among the 700 patients, 32 patients were treated by radical surgery (laparoscopic ureteroneocystostomy) and 579 patients were treated by simple or extensive ureterolysis.^17^, ^18^, ^19^, ^20^, ^21^, ^22^, ^23^, ^24^, ^25^, ^26^, ^27^, ^28^, ^29^ Despite surgical morbidity, their review highlighted the resolution or improvement of symptoms observed in 343 of 379 patients (90.5%). Persistent or recurrent symptoms were observed in 3.9% (95% confidence interval [CI] 0.5%–7.3%) in the ureterolysis group and 0% in the radical surgery group.^16^ The scarcity of data in the literature and the variety and non‐consensual status of UTE management lead to each team developing their own protocols. Persistent or recurrent symptoms were observed in 3.9% (95% CI 0.5%–7.3%) in the ureterolysis group and 0% in the radical surgery group.^16^

In view of the incomplete knowledge on this subject, the present study aims (1) to report the incidence of UTE among patients with deep endometriosis, (b) to analyze the surgical procedures, (c) to report the rate of intra‐ and postoperative complications, and (4) to report the recurrence rate in a French expert center of endometriosis.

## 2 MATERIALS AND METHODS

### 2.1 Population

Data were collected from a prospective monocentric database covering a 6‐year period (January 2016 to December 2022), and then retrospectively analyzed at the Tenon Hospital Endometriosis Expert Center (C3E, AP‐HP, Paris, France).

All patients with deep endometriosis undergoing surgery during the study period were screened based on their surgical and pathologic reports. Deep endometriosis referred to lesions infiltrating the peritoneum deeper than 5 mm (not superficial).

Ureteral endometriosis was recently defined by Talreja et al.^30^ as endometriotic lesions involving the overlying peritoneum, uterosacral ligament, or ovary resulting in extrinsic compression of the ureteral wall as well as lesions involving the ureteral mucosa or muscularis. The following definitions were used to describe the urologic procedures. Simple ureterolysis described when the ureter was spotted and dissected to remove endometriosis lesions at distance from the ureter. Difficult ureterolysis described when ureter dissection was mandatory to separate endometriosis lesions from the ureter but without catheterization. Extensive ureterolysis described when a catheterization was required or resection of ureteral segment. The indication was dependent on the aspect of the ureter following the dissection (severe stenosis).

In the present work, patients selected were those requiring at least a partial bladder resection, extensive ureterolysis, and ureterocystoneostomy. Patients included often had associated deep endometriosis lesions such as colorectal endometriosis or large ovarian endometrioma. Patients who underwent solely superficial bladder shaving were not included and patients under 18 years old were excluded.

The research project was approved by the Comité d'Éthique pour la Recherche en Obstétrique et Gynécologie (number 2023–GYN–0904).^31^

### 2.2 Patient management

The diagnostic strategy include an interview to determine symptoms as well as previous treatments and surgeries, followed by a physical examination including speculum and digital vaginal examination.^32^ Preoperative digital rectal examination was performed in the case of suspicion of rectal or parametrial deep endometrioisis.

All patients underwent systematic imaging including transvaginal ultrasonography (TVUS) or magnetic resonance imaging (MRI) with systematic preoperative review during the multidisciplinary endometriosis committee meetings. The multidisciplinary team consisted of gynecologists specialized in gynecologic surgery and assisted reproductive technology, as well as radiologists and urologists. The need for complementary examinations was always discussed in these sessions if appropriate.

**FIGURE 1:**
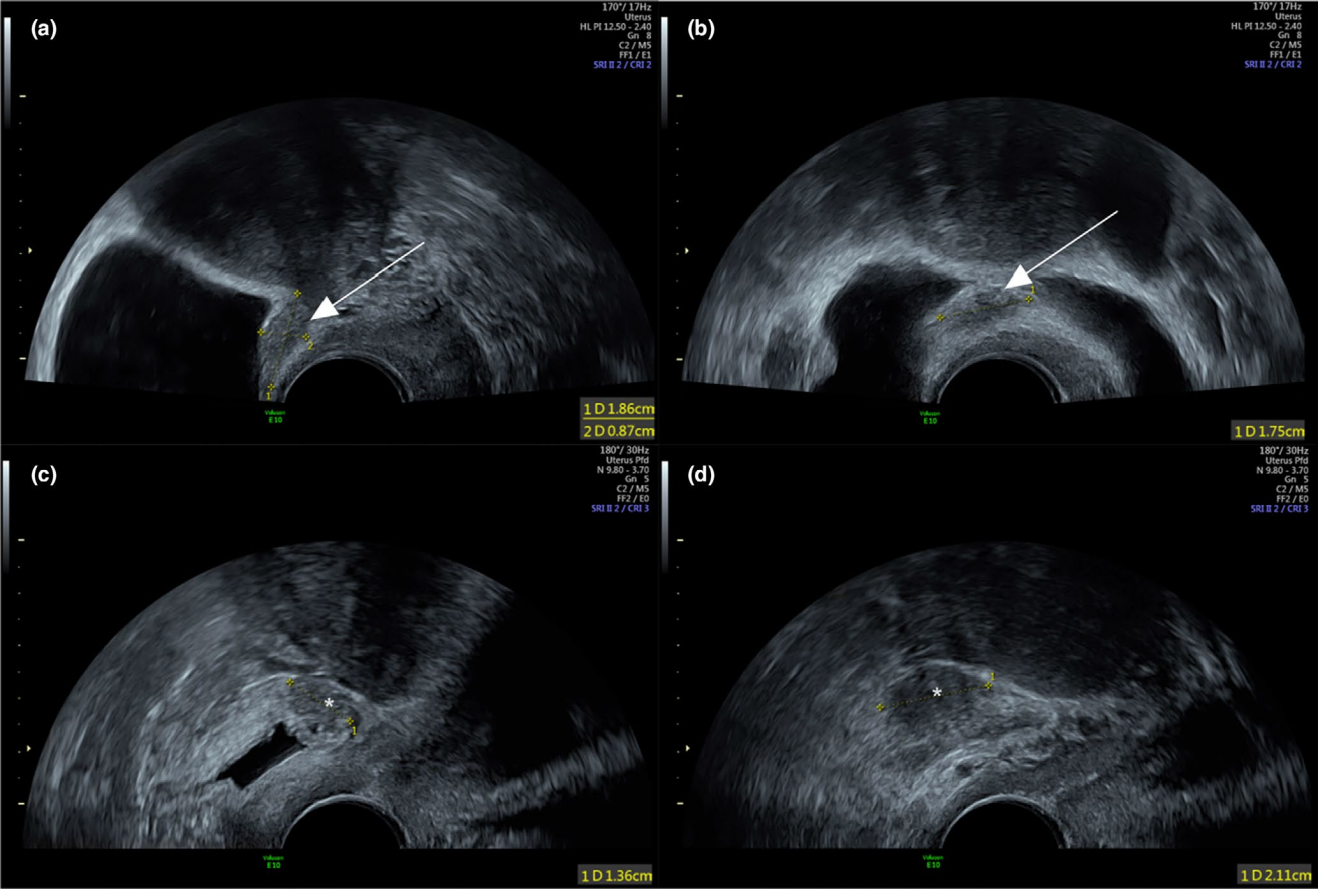
Ultrasound appearance of anterior subperitoneal endometriosis in the vesicouterine pouch. Sagittal (a) and axial (b) endovaginal ultrasound slices showing a 19 × 18 × 9‐mm nodule, isoechoic to the vesicouterine pouch in a 30‐year‐old patient (white arrow). Ultrasound appearance of anterior bladder subperitoneal endometriosis. Sagittal (c) and axial (d) endovaginal ultrasound slices showing a 21 × 14‐mm iso/hypoechoic nodule infiltrating the bladder muscle in a 38‐year‐old patient (white star).

On TVUS, bladder involvement was defined by a thickening of the bladder detrusor muscle, which appears hypoechoic or isoechoic, and can be linear or nodular and associated with the presence of hyperechoic spots or small cysts indicative of an endometriotic lesion (Figure 1).

**FIGURE 2:**
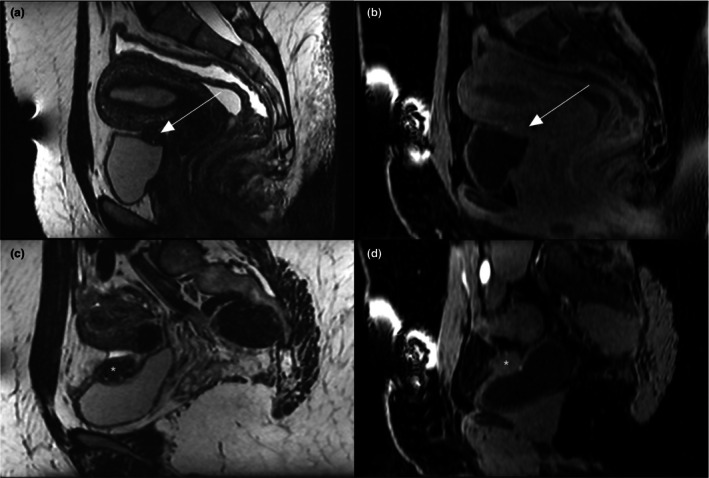
Magnetic resonaonce imaging (MRI) correlation of anterior subperitoneal involvement in the vesicouterine pouch with three‐dimensional (3D) sagittal T2‐weighted sequences (a) and 3D sagittal T1 DIXON WATER‐weighted sequences (b), showing the vesicouterine pouch nodule in T2 hypointensity with hyperintense T1 WATER spots indicative of involvement in the vesicouterine pouch and detrusor in the same 30‐year‐old patient (white arrow). MRI correlation of anterior subperitoneal involvement in the bladder dome with 3D sagittal T2‐weighted sequences (c) and 3D sagittal T1 DIXON WATER‐weighted sequences (d), showing the bladder dome nodule in T2 hypointensity with hyperintense T1 WATER spots indicative of involvement of the detrusor in the vesical dome in the other 38‐year‐old patient (white star).

**FIGURE 3:**
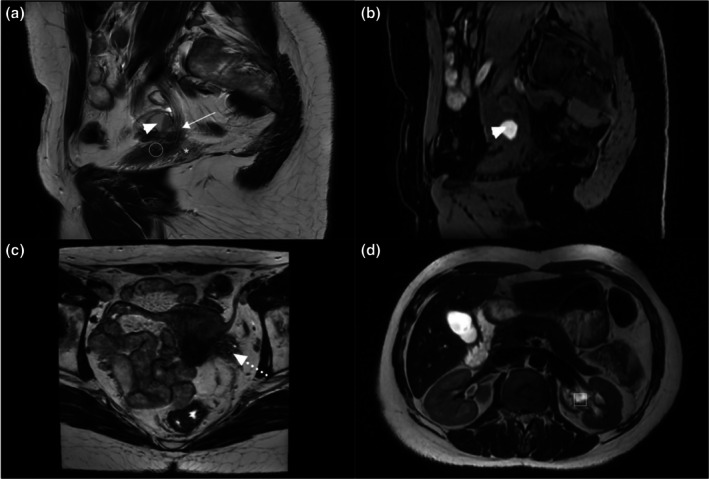
A 32‐year‐old patient with endometriosis involving lateral subperitoneal structures. 3D T2‐weighted magnetic resonance imaging (MRI) sequences, sagittal slices (a) showing ureteral involvement (white arrow) with an intraluminal JJ stent, adjacent parametrial involvement (white star), and bladder involvement (white circle). Notably, an adjacent endometrioma (white arrowhead) with hyperintense signal corresponding to the T1 WATER‐weighted MRI sequences, sagittal slices (b) (white arrowhead). Corresponding involvement in 3D T2‐weighted sequences, axial slices (dotted white arrow) (c), and dilation of the pyelic cavities of the upstream left kidney (white rectangle) (d).

On MRI, bladder endometriosis was diagnosed by the presence of a nodule or mass located in the superior, posterior, or lateral bladder wall, appearing in hypo‐ or isointense T2 signal with hyperintense spots in T1 with fat suppression.^33^ Regarding bladder involvement, the extent is reported according to three different levels within the bladder wall: (1) anterior two‐thirds of the dome; (2) the posterior third of the dome at the level of the vesicouterine pouch; and (3) the bladder base^34^ (Figure 2). Ureteral involvement was assessed in the lateral compartment, especially in cases of lateral parametrial involvement associated with ureteral compression, ureterohydronephrosis, or even renal destruction^35^ (Figure 3). All MRI examinations included slices of kidney using axial T2‐weighted lumbo‐pelvic imaging to evaluate an associated dilatation of pyelocaliceal cavities. For patients with a suspicion of bladder endometriosis, a preoperative cystoscopy was recommended to evaluate the size and the location of endometriosis as well as the distance between the lower limit of the nodule and the ureters.

In case of severe ureterohydronephrosis, renal scintigraphy was performed to decide between conservative surgery and nephrectomy.

Based on national and international guidelines, surgery was recommended in cases of failure of the initial medical treatment, recurrence, or multiorgan involvement and hydronephrosis.^36^, ^37^ All surgical procedures aimed to completely remove the disease.^36^ All surgeries were performed using a laparoscopic approach and from 2019, most procedures were robotically assisted by the Da Vinci Xi (Intuitive).^38^

Preoperative ureteral JJ stenting is commonly performed to reduce the risk of intraoperative ureteral injury and to facilitate the surgical procedure. The stent is placed in the ureter between the kidney and the bladder under endoscopic and radiographic control. This procedure was not systematic and was left to the discretion of the surgeons. Urologists were always involved during surgery and assessed the necessary procedures for the patients (JJ stent, ureterocystostomy).

Postoperative complications were assessed using the Clavien‐Dindo classification system as low grade (Grade I–II) or high grade (Grade IIIA and IIIB–IV).^39^, ^40^ Major complications were those requiring either radiologic or surgical drainage or the need for surgical revision Radiologic drainage refers in our work to the use of imaging techniques to guide the placement of a catheter for draining fluids or abscesses from the body.

In the postoperative setting, voiding dysfunction was defined as the need for self‐catheterization following surgery.^41^

### 2.3 Statistical analysis

Databases were managed using Excel (Microsoft Corporation, Redmond, WA, USA) and statistical analyses were performed using R software (3.3.1 version, available online).

Differences between the main categorical variables were assessed using the χ^2^ test or Fisher exact test, depending on the number of participants. Quantitative variables were compared using Student *t* test, the Wilcoxon rank‐sum test, analysis of variance, or the Kruskal‐Wallis test. For all analyses performed, a value of *p* < 0.05 was considered significant.

In the present study, we distinguished three groups based on the location of endometriosis in the urinary tract. Bladder endometriosis refers to isolated bladder lesions. Ureteral endometriosis refers to patients requiring extensive ureterolysis with JJ stent or ureterocystoneostomy. Mixed endometriosis involves patients with both bladder and extensive ureterolysis.

## 3 RESULTS

### 3.1 Population

During the inclusion period, 923 patients underwent surgical management for deep endometriosis. Among them, 99 (11%) had urinary tract involvement: 13 (13%) with isolated bladder lesions, 56 (57%) with extensive ureterolysis, and 30% with mixed lesions.

**FIGURE 4:**
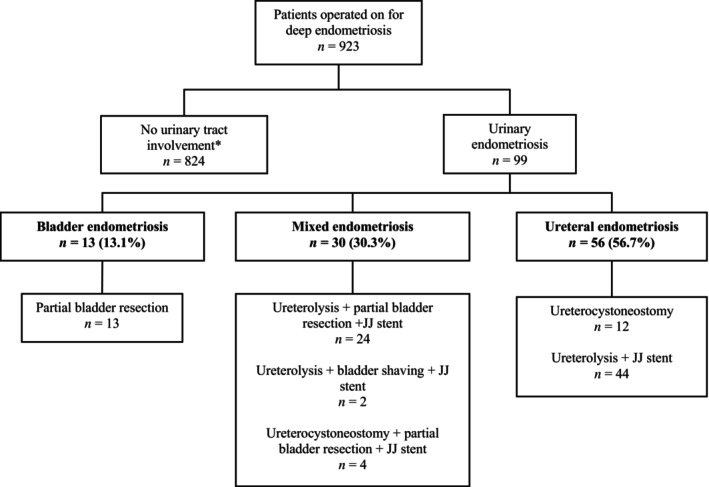
Flow chart of the study; *according to stringent criteria afore mentioned.

The flow chart (Figure 4) displays the distribution of lesions and the procedures performed in our cohort.

The main clinical features are displayed in Table 1. Median age at surgery was 34 years (31–40 years). Most patients were nulliparous (58%). Forty patients (40%) had at least one previous surgery for endometriosis and 42 (42%) were infertile. One‐third of patients experienced lower urinary tract symptoms (34%). Lower urinary tract symptoms were more often associated with bladder involvement (26/34, 76.5%; *p* < 0.001).

**TABLE 1:** Demographic and clinical characteristics of patients.^a^

Characteristic	Total (*n* = 99)	Bladder endometriosis (*n* = 13)	Ureteral endometriosis (*n* = 56)	Mixed endometriosis (*n* = 30)	*p* value
Age, y	34 (31–40)	31 (29–35)	35 (31–41.2)	34 (31–39)	0.24
BMI	24 (20.8–27.4)	21.1 (19.3–22.1)	24.6 (22.3–27.6)	23 (20.4–27.7)	0.04
Parity					0.14
0	57 (58)	9 (69)	26 (46)	22 (73)	
≥1	42 (42)	4 (31)	30 (54)	8 (27)	
Prior endometriosis surgery	40 (40)	1 (8)	28 (50)	11 (37)	0.02
Patients with infertility	42 (42)	4 (31)	27 (48)	11 (37)	0.35
Symptoms					
No symptoms	5	2	2	1	0.14
Dysmenorrhea	80 (81)	10 (77)	45 (80)	25 (83)	1
Dyspareunia	56 (57)	5 (39)	35 (63)	16 (53)	0.17
Chronic pelvic pain	45 (46)	3 (23)	30 (54)	12 (40)	0.09
Pain on micturition	18 (18)	6 (46)	2 (4)	10 (33)	<0.001
Dyschezia	13 (13)	1 (8)	6 (11)	6 (20)	0.52
Pain on defecation	29 (29)	2 (15)	17 (30)	10 (33)	0.5

Abbreviation: BMI, body mass index (calculated as weight in kilograms divided by the square of height in meters).

^a^ Data are presented as median (range) or number (percentage).

Indications for surgery were pelvic pain in 68 patients (69%), infertility in three patients (3%), and both in 23 cases (23%). Five patients were completely asymptomatic but required surgery for hydronephrosis.

### 3.2 Diagnostic and pretherapeutic assessment

Sixteen patients (16%) had no sign of deep endometriosis following vaginal examination. Forty‐seven patients (48%) had vaginal or rectovaginal septum involvement, and 12 patients (12%) had rectal involvement (Table 2). Patients with clinical parametrial involvement had ureteral lesions significantly more often than those with bladder or mixed lesions (19/56 versus 5/43, *p* < 0.001).

**TABLE 2:** Deep endometriosis location at clinical examination and on imaging.^a^

Location	Total (*n* = 99)	Bladder endometriosis (*n* = 13)	Ureteral endometriosis (*n* = 56)	Mixed endometriosis (*n* = 30)	*p* value
Endometriosis locations on clinical examination					
Torus	68 (69)	7 (54)	48 (86)	13 (43)	<0.001
Uterosacral ligament	68 (69)	7 (54)	47 (84)	14 (47)	<0.001
Parametrium	24 (24)	2 (16)	19 (34)	3 (10)	0.04
Vagina	47 (48)	6 (46)	29 (52)	12 (40)	0.44
Rectum	34 (34)	3 (23)	26 (46)	5 (17)	0.01
Endometriosis locations on imaging examinations					
Torus	50 (51)	7 (54)	34 (61)	9 (30)	<0.001
USL	49 (50)	7 (54)	33 (59)	9 (30)	<0.001
Parametrium	27 (27)	2 (16)	19 (34)	6 (20)	0.17
Ureteral	16 (16)	0	13 (23)	3 (10)	0.05
Bladder	23 (23)	13 (100)	0	16 (53)	<0.001
Kidney	8 (8)	0	7 (13)	1 (3)	0.23
Rectum	30 (30)	3 (23)	21 (38)	6 (20)	0.69
Other digestive locations	2 (2)	0	1 (2)	1 (3)	1
Preoperative hormonal treatment	40 (40)	4 (31)	28 (50)	8 (27)	0.09
Preoperative fertility preservation	5 (5)	0	5 (9)	0	0.25

Abbreviation: USL, uterosacral ligament.

^a^ Data are presented as number (percentage).

Pelvic MRI including renal areas was performed in 81 patients (82%). Two patients only had an ultrasound and missing data on the preoperative workup occurred in 17 cases.

Rectal echoendoscopy was performed in 39 patients (39%) when rectal involvement was detected on MRI. A preoperative entero‐MRI to detect multifocal and multicentric bowel endometriosis was performed in 33 patients (33%) with confirmed rectal involvement. Thirty patients (30%) underwent diagnostic cystoscopy. The mean size of bladder nodule endometriosis was 30 mm (25–30 mm). The mean distance between the ureteral meatus and the inferior limit of the bladder nodule was 20 mm (15–30 mm).

Gonadotropin‐releasing hormone agonist treatment was prescribed for 3 months before surgery in 40 (40%) cases.

According to our results, following the review of images from MRI scans by our radiologists: MRI has higher sensitivity (68.4%) and specificity (96.6%) compared with clinical diagnosis (sensitivity 37.9%, specificity 94.4%), with the κ coefficient also being higher for MRI (κ = 0.33) than for clinical diagnosis (κ = 0.26) but remaining limited.

### 3.3 Surgical management

Conventional laparoscopy was performed on 82 patients (83%), 11 robot‐assisted laparoscopy (Table 3). Six patients underwent a conversion to laparotomy for severe intraoperative bleeding including two with isolated bladder endometriosis, two with ureteral endometriosis, and two with mixed endometriosis. Hysterectomy was performed in 33 patients, who were older than those who underwent conservative surgery (median 40 years [37–43 years] versus 32 years [29.2–34 years]; *p* < 0.001): two in the bladder group, 25 in the ureteral group, and six in the mixed group.

**TABLE 3:** Surgical intraoperative data.^a^

	Total (*n* = 99)	Bladder endometriosis (*n* = 13)	Ureteral endometriosis		Mixed endometriosis (*n* = 30)
			Ureterolysis (*n* = 44)	Ureterovesical reimplantation (*n* = 12)	
Surgical route					
Laparoscopy	82 (83)	10 (77)	39 (89)	6 (50)	27 (90)
Robot‐assisted laparoscopy	11 (11)	1 (8)	3 (7)	6 (50)	1 (3)
Laparotomy conversion	6 (6)	2 (15)	2 (5)	0	2 (7)
Urinary tract gestures					
Preoperative JJ stent	20 (20)	1 (8)	6 (14)	6 (50)	7 (23)
Bladder shaving	2 (2)	0	0	0	2 (7)
Partial bladder resection	41 (41)	13 (100)	0	0	28 (93)
Radical cystectomy	0	0	0	0	0
Ureterolysis	86 (87)	0	44 (100)	12 (100)	30 (100)
Ureteral resection with end‐end termino‐terminal anastomosis	0	0	0	0	0
Ureteral resection uretero‐vesical reimplantation	16 (16)	0	0	12 (100)	4 (13)
Intraoperative JJ stent	77 (78)	1 (8)	44 (100)	12 (100)	20 (67)
Nephrectomy	0	0	0	0	0
Digestive tract procedures					
Rectal shaving	19 (19)	2 (15)	8 (18)	4 (33)	5 (17)
Discoid resection	10 (10)	1 (8)	8 (18)	0	1 (3)
Segmental colorectal resection	39 (39)	1 (8)	22 (50)	6 (50)	10 (33)
Protective stomia	24 (24)	0	13 (30)	5 (42)	6 (20)
Ileocecal resection	13 (13)	0	8 (18)	0	3 (10)
Bowel resection	6 (6)	0	4 (9)	0	2 (7)
Other procedures					
USL resection	75 (76)	6 (46)	40 (91)	12 (100)	17 (57)
Parametrectomy	62 (63)	1 (8)	35 (80)	12 (100)	14 (47)
Douglassectomy	23 (23)	0	13 (30)	4 (33)	6 (20)
Hysterectomy	33 (33)	2 (15)	19 (43)	6 (50)	6 (20)
Partial colpectomy	27 (27)	1 (8)	17 (39)	4 (33)	5 (17)

Abbreviation: USL, uterosacral ligament.

^a^ Data are presented as number (percentage).

In our cohort, median operating time was 260 minutes (183–336 minutes) and median intraoperative blood loss was 150 mL (100–400 mL). Five patients required intraoperative blood transfusions.

The distribution of surgical procedures according to the groups is summarized in Table 3.

### 3.4 Surgical outcomes

The main surgical outcomes are displayed in Table 4. Median hospital stay was 8 days (5–12 days). In the absence of postoperative complications, the median hospital stay was 6 days (3–8 days) versus 10 days (7–13.5 days) in case of complication (*p* < 0.001).

**TABLE 4:** Intraoperative and postoperative data.^a^

	Total (*n* = 99)	Bladder endometriosis (*n* = 13)	Ureteral endometriosis (*n* = 56)	Mixed endometriosis (*n* = 30)	*p* value
Abdominal drain	77 (78)	6 (46)	51 (91)	20 (67)	<0.001
Postoperative data					
Hospital stay, d	8 (5–12)	4 (3–10)	8 (7–12)	7 (4–13)	0.76
Postoperative voiding dysfunction requiring self catherization					
Self‐catheterization	17 (17)	1 (8)	12 (21)	2 (7)	0.56
Duration of self‐catheterization, wk	20 (7.5–75)	0	20 (13–92)	12 (8.5–36)	0.33
Voiding dysfunction >1 mo	7 (7)	0	5 (9)	2 (7)	0.86
Complications					0.35
Grade I	11 (11)	1 (8)	6 (11)	4 (13)	
Grade II	30 (30)	7 (54)	18 (32)	5 (17)	
Grade III	15 (15)	1 (8)	11 (20)	3 (10)	
IIIa	2 (2)	0	1 (2)	1 (3)	
IIIb	13 (13)	1 (8)	10 (18)	2 (7)	
Grade IV	2 (2)	0	1 (2)	1 (3)	

^a^ Data are presented as median (range) or number (percentage).

The overall complication rate was 59% (58/99) including 41 patients with low grade complications and 17 patients with severe complications. The most frequent complications were low grade, including urinary infection (three patients) or pyelonephritis (eight patients).

For the 17 patients experiencing a high‐grade complication, data are summarized in Table 5. Among them, the rate of Grade IIIa complication or greater was higher in patients with previous surgery for endometriosis (64.7% versus 35.4%, *p* = 0.024). A trend for an association between obesity and the risk of severe postoperative complications was noted (*p* = 0.084).

**TABLE 5:** Summary of data from patients with Clavien‐Dindo complications ≥3a.

No.	Characteristics	Surgical route	Postoperative data	Complication
1	35 years old BMI: NA History of laparotomy (ovariectomy) Symptoms: Pain, metrorrhagia	Hysterectomy Torus, USL Rectal shaving Parametrectomy Partial bladder resection Ureterolysis	Duration before removal of urinary catheter: 5 days	Bladder suture failure resulting in pelvic peritonitis 1 month postoperative Surgical revision by laparoscopy: bladder suture and peritoneal washing
2	33 years old BMI: NA History of laparoscopy for endometriosis (cystectomy for endometrioma) and appendicectomy Symptoms: pain, infertility	Torus, USL Rectal shaving Endometrioma fenestration Mixed endometriosis Ureterolysis Partial bladder resection Perioperative JJ stent Parametrectomy Salpingectomy Perioperative complication: left external iliac vein injury Operative time: 390 min Estimated blood loss: 400 mL	Length of hospital stay: 18 days Discharge with urinary catheter	Pelvic abscess Replacement of JJ stents and abscess drainage
3	43 years old BMI: 36.6 History of laparoscopy (cholecystectomy) and cesarean section Symptoms: Dysmenorrhea, dyspareunia	Torus, USL Discoid resection Endometrioma fenestration Hysterectomy Ureteral endometriosis Ureterolysis Perioperative JJ stent Parametrectomy Estimated blood loss: 250 mL	Length of time before removal of urinary catheter: 5 days Self‐catheterization learning Length of hospital stay: 12 days	Vesicovaginal fistula Surgical revision by laparoscopic
4	36 years old BMI: 28.3 History of laparoscopy for endometriosis (excision of rectovaginal septum lesion and colpectomy) Symptoms: Dyspareunia	Torus, USL Colorectal resection Ileocecal resection Colpectomy Hysterectomy Ureteral endometriosis Ureterolysis Perioperative JJ stent Parametrectomy Protective stomia Estimated blood loss: 500 mL	Length of hospital stay: 23 days	Left ureteral fistula
5	42 years old BMI: 16.9 History of laparoscopy for endometriosis (torus, USL and colorectal resection) Symptoms: pelvic pain	Torus, USL Colorectal resection Discoid resection Hysterectomy Ureteral endometriosis Ureterolysis Perioperative JJ stent Parametrectomy Estimated blood loss: 300 mL	Length of time before removal of urinary catheter: 6 days Length of hospital stay: 8 days	Pyelonephritis due to misplaced JJ stent and urinary leakage
6	33 years old BMI: NA History of laparoscopy for endometriosis (ethanol sclerotherapy endometrioma) Symptoms: pelvic pain, infertility	Torus, USL Colorectal resection Colpectomy Ureteral endometriosis Ureterolysis Perioperative JJ stent Parametrectomy Protective stomia Estimated blood loss: 700 mL	Length of time before removal of urinary catheter: 5 days Postoperative voiding dysfunction, learned self‐catheterization Length of hospital stay: 12 days	10‐cm presacral abscess, radiologically drained
7	31 years old BMI: 26 Smoking History of laparotomy for endometriosis (salpingectomy) Symptoms: pelvic pain and uretero‐hydronephrosis	Torus, USL Colorectal resection Hysterectomy Ureteral endometriosis Ureterolysis Ureteral reimplantation Perioperative JJ stent Parametrectomy Protective stomia Operative time: 480 min Estimated blood loss: 500 mL	Length of time before removal of urinary catheter: 5 days Length of hospital stay: 14 days	Pyelonephritis due to misplaced JJ stent
8	30 years old BMI: 20.8 Symptoms: infertility	USL Rectal shaving Ureteral endometriosis Ureterolysis Perioperative JJ stent Douglassectomy Estimated blood loss: 50 mL	Length of time before removal of urinary catheter: 1 days Length of hospital stay: 7 days	Hemoperitoneum
9	30 years old BMI: 18.4 History of laparoscopy for endometriosis Symptoms: dysmenorrhea, dyspareunia	Torus, USL Colorectal resection Colpectomy Mixed endometriosis Ureterolysis Partial bladder resection Perioperative JJ stent Parametrectomy Protective stomia Estimated blood loss: 500 mL	Length of time before removal of urinary catheter: 15 days Postoperative voiding dysfunction, learned self‐catheterization Length of hospital stay: 21 days	Sepsis complicated pyelonephritis Change of JJ stent at postoperative day 6
10	43 years old BMI: 28 History of laparoscopy for endometriosis (hysterectomy, colorectal resection, parametrectomy, colpectomy) and laparotomy (myomectomy) Symptoms: pelvic pain	Laparotomy: Mixed endometriosis Ureterolysis Ureteral reimplantation Partial bladder resection Perioperative JJ stent Operative time: 270 min Estimated blood loss: 200 mL	Length of time before removal of urinary catheter: 14 days Length of hospital stay: 10 days Readmission	Sepsis complicated pyelonephritis due to misplaced JJ stent Cardiogenic shock due to septic myocarditis
11	30 years old BMI: 20.6 History of laparotomy (appendiceal peritonitis with right colectomy and upper genital tract infections with adnexectomy) and laparoscopy (ovarian cystectomy) Symptoms: pain	Torus, USL Rectal shaving Colpectomy Ureteral endometriosis Ureterolysis Perioperative JJ stent Parametrectomy Estimated blood loss: 150 mL	Length of time before removal of urinary catheter: 9 days Length of hospital stay: 9 days Readmission	Vaginal cuff dehiscence with vaginal bleeding on day 15
12	31 years old BMI: 27.2 History of laparoscopy for endometriosis (cholecystectomy and ovarian cystectomy) Symptoms: pain, infertility	Laparoconversion Torus, USL Colorectal resection Colpectomy Ureteral endometriosis Perioperative JJ stent Parametrectomy Protective stomia Operative time: 419 min Estimated blood loss: 700 mL Perioperative transfusion	Duration before removal of urinary catheter: 7 days Postoperative voiding dysfunction, learned self‐catheterization Length of hospital stay: 13 days	Sepsis complicated pyelonephritis Change of JJ stent at postoperative day 2
13	28 years old BMI: 30.4 History of laparoscopy for endometriosis (hysterectomy, partial colpectomy, parametrectomy, rectal shaving, JJ stent)	Mixed endometriosis Ureterolysis Ureteral reimplantation Partial bladder resection Perioperative JJ stent Parametrectomy Operative time: 480 min Estimated blood loss: 250 mL	Length of hospital stay: 17 days	Surgical revision on day 1 for incisional hernia. Day 3: vesicovaginal fistula, installation of ureteral stent Day 13: change ureteral stent by JJ stent Readmission for pyelonephritis
14	33 years old BMI:33.1 History of laparoscopy for endometriosis (endometrioma fenestration, rectal shaving, douglassectomy) Symptoms: Pain	Torus, USL Colorectal resection Partial colpectomy Ureteral endometriosis Ureterolysis Perioperative JJ stent Parametrectomy Protective stomia Operative time: 242 min Estimated blood loss: 150 mL	Length of time before removal of urinary catheter: 4 days Length of hospital stay: 10 days	Ureteral fistula with ureteral stenosis with downstream stenosis Day 1: nephrostomy Ureterovesical reimplantation simultaneous with stoma closure Incisional hernia 8 months postoperative
15	33 years old BMI: 22.7 History of laparoscopy and laparotomy for endometriosis ovarian cystectomy, salpingectomy, peritoneal endometriosis Symptoms: pain, metrorrhagia	Torus, USL Discoid resection Hysterectomy Ureteral endometriosis Ureterolysis Perioperative JJ stent Parametrectomy	Length of time before removal of urinary catheter: 6 days Length of hospital stay: 8 days Readmission in urology to change JJ stent	Stenosis of the distal ureter with placement of a new JJ stent and right adnexectomy, ureterolysis, and right ureterovesical reimplantation.
16	29 years old BMI: 23.8 History of JJ stent placement Symptoms: sciatica	Torus, USL Discoid resection Ureteral endometriosis Ureterolysis Perioperative JJ stent Operative time: 260 min	Length of time before removal of urinary catheter: 7 days Length of hospital stay: 12 days	Hemorrhage from discoid resection. Surgical revision: segmental resection with segmental anastomosis
17	38 years old BMI:27.3 Symptoms: pain	Torus, USL Colorectal resection Hysterectomy Partial colpectomy Ureteral endometriosis Ureterolysis Ureteral reimplantation Perioperative JJ stent Parametrectomy Protective stomia Operative time: 420 min Estimated blood loss: 250 mL	Length of time before removal of urinary catheter: 15 days Length of hospital stay: 17 days	Bladder blood clot evacuation and pulmonary embolism

Abbreviations: BMI, body mass index (calculated as weight in kilograms divided by the square of height in meters); NA, not available; USL uterosacral ligament.

Five patients (5%) had anastomosis (vesical or ureteroneocystostomy) related complications—four patients (4%) needed a postoperative change of the JJ stent and one patient (1%) required bladder blood clot removal. Five patients required surgical revision: three patients (3%) had bleeding and two patients (2%) had intra‐abdominal abscesses drained by laparoscopy.

Seventeen patients (78%) experienced postoperative voiding dysfunction requiring self‐catheterization, including seven with persistent voiding dysfunction of longer than 1 month. Data were missing for two patients.

At the 1‐month postoperative visit, 85% of the patients had no need to take painkillers and were completely relieved. Among the remaining patients, two still required level 2 analgesics (morphine equivalent).

During follow up (median 6 months [1–12 months]), three patients experienced recurrence of their endometriosis (3%): two at 1 year and one at 5 years postoperatively.

## 4 DISCUSSION

The present study on UTE from an expert center in endometriosis confirms that isolated bladder involvement is rare, whereas ureteral involvement is often associated with deep endometriosis lesions. Conventional or robot‐assisted laparoscopy are feasible and safe approaches to manage UTE, but expose women to severe complications. Given the high rates of peri‐ and postoperative complications, our study highlighted the risk factors of complications contributing to a better decision making process.

In our cohort, using stringent criteria, urinary endometriosis was observed in 11% of women, suggesting that the incidence of UTE is probably underestimated. This rate could be explained by the recruitment being at an expert center. However, similar rates of UTE were reported by Knabben et al. Indeed, in their experience, among the 213 patients with deep endometriosis, ureteral involvement was described in 49.8% of cases and bladder involvement in 7.5%. In the cohort of Froc et al., 150 patients (64.6%) had ureteral involvement associated or not with bladder involvement and 106 patients (45.7%) presented with bladder involvement associated or not with a ureteral involvement.^11^ The discrepancy across studies could also be explained by recruitment bias.

For management of UTE, a comprehensive pretherapeutic evaluation is required. Our results underline both the low sensitivity of physical examination and MRI despite a high specificity. These considerations underline the crucial contribution of multidisciplinary endometriosis committees to planning surgery and informing patients on the surgical risks.

In the present study, most patients (94%) underwent conventional or robot‐assisted laparoscopy and 6% underwent a laparotomy conversion. In contrast, in 2021, the FRIENDS group published data from 31 French centers involving 232 patients with UTE.^42^ Laparotomy was chosen as the initial surgical approach in 26% of cases, particularly in situations involving mixed locations (*p* = 0.007), when ureteral resection was required (*p* = 0.02), or when the procedure was performed jointly with urologists (*p* = 0.004). In our series, no conversion was related to the presence of a urologist, likely due to the high level of experience of our urologists in both laparoscopy, robotic surgery, and endometriosis. Our rate of laparotomy conversion is in agreement with previous studies (1.4%–3.9%) with a nine‐fold increase in the case of associated bladder and ureteral lesions.^16^, ^42^, ^43^ Our laparotomy conversion is in agreement with previous studies (1.4%–3.9%) with a nine‐fold increase in case of associated bladder and ureteral lesions.^16^, ^42^, ^43^ Regarding the robotic approach, Raimondo et al. comparing conventional and robot‐assisted laparoscopy underlined the discrepancies between series depending on the experience of the center and the sample size.^44^ Di Maida et al. reported the largest series comparing robotic and conventional laparoscopy in UTE, showing a lower complication rate in the robotic group (*p* = 0.02) with similar functional and recurrence rates (*p* = 0.70).^45^

Another crucial issue is to determine the risk of postoperative complications. In our cohort, using Clavien‐Dindo classification, Grade I–II and Grade III and above complications occurred in 41% and 17% of patients, respectively. Complications requiring revision surgery or interventional radiology were observed in 17 patients (17%). Among them, the rate of Grade IIIa or above complications was higher in patients with previous surgery for endometriosis (64.7% versus 35.4%, *p* = 0.024). Five patients (5%) had an anastomotic complication imposing a change of JJ stent in four patients and one undergoing bladder clot evacuation. In addition, a second surgery was required for bleeding in three patients and for pelvic abscess in two patients. In our series, the rate of postoperative voiding dysfunction was 17.2%, and 7% lasted more than 1 month. The rate of voiding dysfunction varied according to surgery; 7.7% in the bladder group and 16.3% in the ureteral and mixed groups. Our rate of voiding dysfunction is consistent with previous studies,^46^ depending on the presence of parametrial involvement and history of surgery for endometriosis.

To predict the risk of postoperative complications, recently the dPEI (Deep Pelvic Endometriosis Index) classification has been proposed and subsequently validated.^47^ The dPEI is a standardized classification system that assesses the extent and severity of deep pelvic endometriosis based on MRI findings to help predict surgical complexity and guide clinical decision making. This classification correlates disease extension with the risk of severe complications (Clavien‐Dindo Grade IIIa or above). Patients with severe disease (deep endometriosis detected in more than five compartments) are 3.6 times more likely to experience severe complications according to the Clavien‐Dindo classification (*p* = 0.004).

A crucial concern in UTE is to determine which surgical procedure to perform. In this work, deep infiltrating urinary endometriosis referred to lesions that extended to the urinary system or complicating central lesions. For bladder endometriosis, a consensus exists on recommending a partial bladder resection.^15^ In our cohort we limited the risk of bias as patients requiring a bladder shaving were excluded. Indeed, under the term bladder shaving, some authors refer to the excision of the serosa alone whereas other refer to partial or total excision of the muscular layer without opening the mucosa. Similarly, the comparison of different cohorts reporting on ureteral endometriosis is complex as no consensus exists on the definition of extensive ureterolysis, posing also an issue on surgical fees. Hence, in the current study, we voluntarily defined extensive ureterolysis when endometriosis lesions imposed a ureter dissection with at least the requirement of a JJ stent. Indeed, simple ureterolysis is a systematic procedure during deep endometriosis excision, even in cases of lesions restricted to uterosacral ligaments, which is the most frequent location of deep endometriosis.^33^ As in the present study, although no clear definition of extensive ureterolysis is propose Donval et al.,^43^ in their retrospective study of 920 patients with deep endometriosis that included 307 patients (42.4%) with a complex ureteral procedure, observed that the main risk factors for a complex ureteral procedure were older age (*p* = 0.036), a previous surgery for endometriosis (*p* < 0.01), the presence of rectosigmoid involvement (*p* < 0.001), rectovaginal septum endometriosis (*p* < 0.01), and a ureteral dilation on MRI (*p* < 0.001). However, in their work, complex ureteral procedures included patients with ureteral “shaving” up to the adventis, leading to a high incidence of complex ureteral procedures up to 30%. To overcome the challenge to compare surgical UTE reports, we propose a new definition of ureteral surgery to better inform patients, hence contributing to a better decision making process.

**FIGURE 5:**
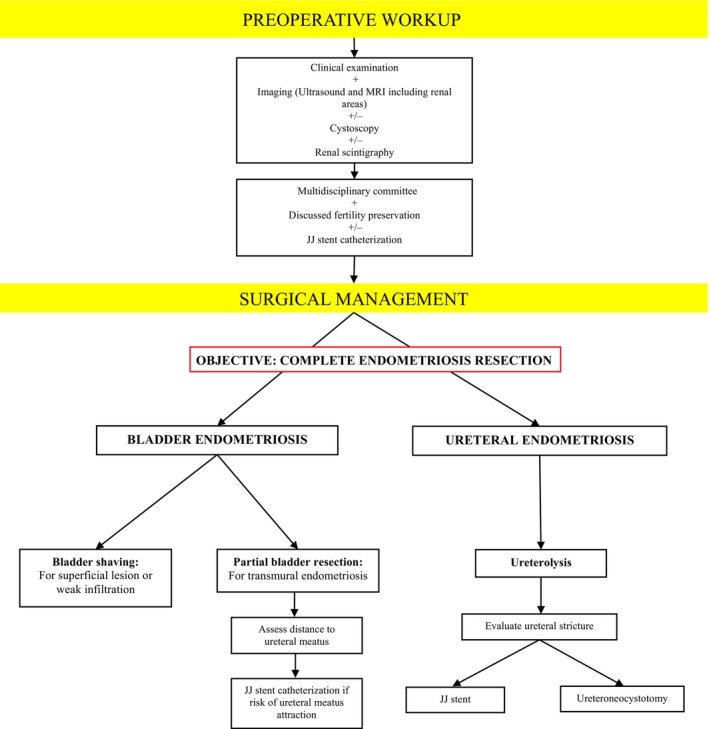
Algorithm for the management of patients with urinary endometriosis.

Another unresolved issue on UTE persists, such as the absence of consensus on the best surgical option between ureterolysis and ureteral resection according to ureteral stricture length as well as the location on the various ureteral segments. Moreover, a debate exists on whether a remaining stricture on the ureter at the end of the surgical gesture could be tolerated. We propose an algorithm for the management of patients with urinary endometriosis (see Figure 5). Patients with confirmed urinary endometriosis should be managed in tertiary referral centers where complementary examinations such as cystoscopy or renal scintigraphy could be performed.

Some limits of the current study deserve to be underlined. First, the retrospective nature introduces potential bias, especially due to missing data. In particular, preoperative workup data were missing. According to Barra et al.,^14^ MRI had a sensitivity of 91% (95% CI 59%–99%) and a specificity of 59% (95% CI 39%–78%). For detection of bladder endometriosis, Maggiore et al.,^12^ reported that pelvic MRI had a pooled sensitivity of 0.64 (95% CI 0.48–0.77) and a pooled specificity of 0.98 (95% CI 0.96–0.99). Second, although our series is large, the sample size remains limited, representing a potential bias. Finally, the long‐term outcomes especially concerning recurrence rate and fertility were lacking because of the limited follow up, necessitating further studies.

In conclusion, our results highlight that UTE can be successfully managed in an experienced center with an acceptable complications rate. Our results highlight the crucial role of the multidisciplinary committee. Finally, our study raises issues on a consensual definition of procedures for deep endometriosis surgery to better evaluate the true complication rates and to improve decision processes.

## AUTHOR CONTRIBUTIONS

CT and ITN contributed to project administration, supervision, and resources; ED and YD contributed to methodology; YD, CDA, LR, AC, YA, SD, FG, and CT acquired the data; YD, CDA, ITN, and ED analyzed the data; and CDA, LR, YD, and ED wrote the original draft. All authors reviewed the manuscript for critical intellectual content.

## CONFLICT OF INTEREST STATEMENT

The authors have no conflicts of interest.

## Data Availability

The data that support the findings of this study are available on request from the corresponding author. The data are not publicly available due to privacy or ethical restrictions.

## References

[1] Zondervan KT , Becker CM , Missmer SA . Endometriosis. N Engl J Med. 2020;382(13):1244‐1256. 32212520 10.1056/NEJMra1810764

[2] Le Moal J , Goria S , Chesneau J , et al. Increasing incidence and spatial hotspots of hospitalized endometriosis in France from 2011 to 2017. Sci Rep. 2022;12(1):6966. 35484205 10.1038/s41598-022-11017-x PMC9050825

[3] Parazzini F , Esposito G , Tozzi L , Noli S , Bianchi S . Epidemiology of endometriosis and its comorbidities. Eur J Obstet Gynecol Reprod Biol. 2017;209:3‐7. 27216973 10.1016/j.ejogrb.2016.04.021

[4] Giudice LC , Kao LC . Endometriosis. Lancet. 2004;364(9447):1789‐1799. 15541453 10.1016/S0140-6736(04)17403-5

[5] Chapron C , Jacob S , Dubuisson JB , Vieira M , Liaras E , Fauconnier A . Laparoscopically assisted vaginal management of deep endometriosis infiltrating the rectovaginal septum. Acta Obstet Gynecol Scand. 2001;80(4):349‐354. 11264611

[6] Koninckx PR , Ussia A , Adamyan L , Wattiez A , Donnez J . Deep endometriosis: definition, diagnosis, and treatment. Fertil Steril. 2012;98(3):564‐571. 22938769 10.1016/j.fertnstert.2012.07.1061

[7] Comiter CV . Endometriosis of the urinary tract. Urol Clin N Am. 2002;29:625‐635. 10.1016/s0094-0143(02)00065-4 12476526

[8] Berlanda N , Vercellini P , Carmignani L , Aimi G , Amicarelli F , Fedele L . Ureteral and vesical endometriosis: two different clinical entities sharing the same pathogenesis. Obstetrical & Gynecological Survey. 2009;64(12):830‐842. 19939297 10.1097/OGX.0b013e3181c4bc3a

[9] Knabben L , Imboden S , Fellmann B , Nirgianakis K , Kuhn A , Mueller MD . Urinary tract endometriosis in patients with deep infiltrating endometriosis: prevalence, symptoms, management, and proposal for a new clinical classification. Fertil Steril. 2015;103(1):147‐152. 25439849 10.1016/j.fertnstert.2014.09.028

[10] Nezhat C , Falik R , McKinney S , King LP . Pathophysiology and management of urinary tract endometriosis. Nat Rev Urol. 2017;14(6):359‐372. 28467398 10.1038/nrurol.2017.58

[11] Froc E , Dubernard G , Bendifallah S , et al. Clinical characteristics of urinary tract endometriosis: a one‐year national series of 232 patients from 31 endometriosis expert centers (by the FRIENDS group). Eur J Obstet Gynecol Reprod Biol. 2021;264:155‐161. 34303076 10.1016/j.ejogrb.2021.06.018

[12] Leone Roberti Maggiore U , Ferrero S , Candiani M , Somigliana E , Viganò P , Vercellini P . Bladder endometriosis: a systematic review of pathogenesis, diagnosis, treatment, impact on fertility, and risk of malignant transformation. Eur Urol. 2017;71(5):790‐807. 28040358 10.1016/j.eururo.2016.12.015

[13] Darai E , Ackerman G , Bazot M , Rouzier R , Dubernard G . Laparoscopic segmental colorectal resection for endometriosis: limits and complications. Surg Endosc. 2007;21(9):1572‐1577. 17342560 10.1007/s00464-006-9160-1

[14] Barra F , Scala C , Biscaldi E , et al. Ureteral endometriosis: a systematic review of epidemiology, pathogenesis, diagnosis, treatment, risk of malignant transformation and fertility. Hum Reprod Update. 2018;24(6):710‐730. 30165449 10.1093/humupd/dmy027

[15] Gourbail L . Recommandations pour la Pratique Clinique – Prise en Charge de l'Endométriose. 2017. 2017.

[16] Cavaco‐Gomes J , Martinho M , Gilabert‐Aguilar J , Gilabert‐Estélles J . Laparoscopic management of ureteral endometriosis: a systematic review. Eur J Obstet Gynecol Reprod Biol. 2017;210:94‐101. 27984749 10.1016/j.ejogrb.2016.12.011

[17] Gabriel B , Nassif J , Trompoukis P , Barata S , Wattiez A . Prevalence and Management of Urinary Tract Endometriosis: a clinical case series. Urology. 2011;78(6):1269‐1274. 21962747 10.1016/j.urology.2011.07.1403

[18] Nezhat C , Nezhat F , Nezhat CH , Nasserbakht F , Rosati M , Seidman DS . Urinary tract endometriosis treated by laparoscopy. Fertil Steril. 1996;66(6):920‐924. 8941055

[19] Donnez J , Nisolle M , Squifflet J . Ureteral endometriosis: a complication of rectovaginal endometriotic (adenomyotic) nodules. Fertil Steril. 2002;77(1):32‐37. 11779587 10.1016/s0015-0282(01)02921-1

[20] Ghezzi F , Cromi A , Bergamini V , Serati M , Sacco A , Mueller MD . Outcome of laparoscopic ureterolysis for ureteral endometriosis. Fertil Steril. 2006;86(2):418‐422. 16764874 10.1016/j.fertnstert.2005.12.071

[21] Frenna V , Santos L , Ohana E , Bailey C , Wattiez A . Laparoscopic management of ureteral endometriosis: our experience. J Minim Invasive Gynecol. 2007;14(2):169‐171. 17368251 10.1016/j.jmig.2006.09.009

[22] Bosev D , Nicoll LM , Bhagan L , et al. Laparoscopic Management of Ureteral Endometriosis: the Stanford University Hospital experience with 96 consecutive cases. J Urol. 2009;182(6):2748‐2752. 19837436 10.1016/j.juro.2009.08.019

[23] Camanni M , Bonino L , Delpiano EM , et al. Laparoscopic conservative management of ureteral endometriosis: a survey of eighty patients submitted to ureterolysis. Reprod Biol Endocrinol. 2009;7(1):109. 19818156 10.1186/1477-7827-7-109 PMC2770480

[24] Mereu L , Gagliardi ML , Clarizia R , Mainardi P , Landi S , Minelli L . Laparoscopic management of ureteral endometriosis in case of moderate‐severe hydroureteronephrosis. Fertil Steril. 2010;93(1):46‐51. 18990377 10.1016/j.fertnstert.2008.09.076

[25] Seracchioli R , Mabrouk M , Montanari G , Manuzzi L , Concetti S , Venturoli S . Conservative laparoscopic management of urinary tract endometriosis (UTE): surgical outcome and long‐term follow‐up. Fertil Steril. 2010;94(3):856‐861. 19481740 10.1016/j.fertnstert.2009.04.019

[26] Seracchioli R , Raimondo D , Di Donato N , et al. Histological evaluation of ureteral involvement in women with deep infiltrating endometriosis: analysis of a large series. Hum Reprod. 2015;30(4):833‐839. 25586785 10.1093/humrep/deu360

[27] Rozsnyai F , Roman H , Resch B , et al. Outcomes of surgical Management of Deep Infiltrating Endometriosis of the ureter and urinary bladder. JSLS. 2011;15(4):439‐447. 22643496 10.4293/108680811X13176785203798 PMC3340950

[28] Soriano D , Schonman R , Nadu A , et al. Multidisciplinary team approach to Management of Severe Endometriosis Affecting the ureter: long‐term outcome data and treatment algorithm. J Minim Invasive Gynecol. 2011;18(4):483‐488. 21777838 10.1016/j.jmig.2011.04.011

[29] Uccella S , Cromi A , Casarin J , et al. Laparoscopy for ureteral endometriosis: surgical details, long‐term follow‐up, and fertility outcomes. Fertil Steril. 2014;102(1):160‐166. 24842674 10.1016/j.fertnstert.2014.03.055

[30] Talreja D , Salunke V , Pande S , Gupta C . Successful management of ureteric endometriosis by laparoscopic ureterolysis – a review and report of three further cases. Arab J Urol. 2018;16(3):342‐349. 30147960 10.1016/j.aju.2018.03.001 PMC6105344

[31] Dabi Y , Thubert T , Fuchs F , Barjat T , Belaisch–Allart J , Ceccaldi PF . How is functionning the ethical review board « Comité d'Ethique pour La Recherche En Obstétrique et Gynécologie » (CEROG) ? J Gynecol Obstet Hum Reprod. 2022;51(4):102352. 35247608 10.1016/j.jogoh.2022.102352

[32] Deffieux X , Rousset‐Jablonski C , Gantois A , et al. Examen pelvien en gynécologie et obstétrique: recommandations pour la pratique clinique. Gynecol Obstet Fertil Senol. 2023;51(6):297‐330. 37258002 10.1016/j.gofs.2023.04.001

[33] Bazot M , Daraï E . Diagnosis of deep endometriosis: clinical examination, ultrasonography, magnetic resonance imaging, and other techniques. Fertil Steril. 2017;108(6):886‐894. 29202963 10.1016/j.fertnstert.2017.10.026

[34] Rousset P , Florin M , Bharwani N , et al. Deep pelvic infiltrating endometriosis: MRI consensus lexicon and compartment‐based approach from the ENDOVALIRM group. Diagn Interv Imaging. 2023;104(3):95‐112. 36404224 10.1016/j.diii.2022.09.004

[35] Bazot M . Imagerie de l'endométriose. Critères diagnostiques.

[36] Collinet P , Fritel X , Revel‐Delhom C , et al. Management of endometriosis. J Gynecol Obstet Hum Reprod. 2018;47(7):265‐274. 29920379 10.1016/j.jogoh.2018.06.003

[37] Becker CM , Bokor A , Heikinheimo O , et al. ESHRE guideline: endometriosis. Hum Reprod Open. 2022;2022(2):hoac009. 35350465 10.1093/hropen/hoac009 PMC8951218

[38] Rabischong B , Botchorishvili R , Bourdel N , et al. Les techniques de préservation nerveuse dans la chirurgie de l'endométriose profonde pour prévenir les séquelles fonctionnelles urinaires et digestives: Modalités techniques et résultats. RPC Endométriose CNGOF‐HAS. Gynecol Obstet Fertil Senol. 2018;46(3):309‐313. 29551299 10.1016/j.gofs.2018.02.031

[39] Clavien PA , Barkun J , De Oliveira ML , et al. The Clavien‐Dindo classification of surgical complications: five‐year experience. Annals of Surgery août. 2009;250(2):187‐196. 10.1097/SLA.0b013e3181b13ca2 19638912

[40] Dindo D , Demartines N , Clavien PA . Classification of surgical complications: a new proposal with evaluation in a cohort of 6336 patients and results of a survey. Ann Surg. 2004;240(2):205‐213. 15273542 10.1097/01.sla.0000133083.54934.ae PMC1360123

[41] Vesale E , Roman H , Moawad G , et al. Voiding dysfunction after colorectal surgery for endometriosis: a systematic review and meta‐analysis. J Minim Invasive Gynecol. 2020;27(7):1490‐1502.e3. 32730989 10.1016/j.jmig.2020.07.019

[42] Philip CA , Froc E , Chapron C , et al. Surgical Management of Urinary Tract Endometriosis: a 1‐year longitudinal multicenter pilot study at 31 French hospitals (by the FRIENDS group). J Minim Invasive Gynecol. 2021;28(11):1889‐1897.e1. 33964459 10.1016/j.jmig.2021.04.020

[43] Donval L , Niro J , Gaillard T , et al. Nomogram for predicting a complex ureteral procedure in pelvic endometriosis surgery. J Minim Invasive Gynecol. 2022;29(5):656‐664. 35063645 10.1016/j.jmig.2022.01.003

[44] Raimondo D , Alboni C , Orsini B , et al. Comparison of perioperative outcomes between standard laparoscopic and robot‐assisted approach in patients with rectosigmoid endometriosis. Acta Obstet Gynecol Scand. 2021;100(9):1740‐1746. 33999408 10.1111/aogs.14170 PMC8453718

[45] Di Maida F , Mari A , Morselli S , et al. Robotic treatment for urinary tract endometriosis: preliminary results and surgical details in a high‐volume single‐institutional cohort study. Surg Endosc Juill. 2020;34(7):3236‐3242. 10.1007/s00464-020-07502-x 32170566

[46] Imboden S , Bollinger Y , Härmä K , et al. Predictive factors for voiding dysfunction after surgery for deep infiltrating endometriosis. J Minim Invasive Gynecol. 2021;28(8):1544‐1551. 33476749 10.1016/j.jmig.2021.01.009

[47] Thomassin‐Naggara I , Monroc M , Chauveau B , et al. Multicenter external validation of the deep pelvic endometriosis index magnetic resonance imaging score. JAMA Netw Open. 2023;6(5):e2311686. 37140921 10.1001/jamanetworkopen.2023.11686 PMC10160872

